# Impact of lung cancer screening on the workload of radiology professionals

**DOI:** 10.1186/s13244-026-02449-4

**Published:** 2026-11-12

**Authors:** Elizabeth Wai Yee Tong, Emily Nischwitz, Anna Kerpel-Fronius, Colin Jacobs, Steven Schalekamp, Roberta E. Ledda, Joanna Moes-Sosnowska, Joanna Chorostowska-Wynimko, Oyunaa von Stackelberg, Helmut Prosch, Hans-Ulrich Kauczor, Viktoria Palm

**Affiliations:** 1 Clinic for Diagnostic and Interventional Radiology (DIR), Heidelberg University Hospital, Heidelberg, Germany; 2 Translational Lung Research Center (TLRC), German Center for Lung Research (DZL), University of Heidelberg, Heidelberg, Germany; 3 Diagnostic and Interventional Radiology with Nuclear Medicine, Thoraxklinik Heidelberg, Heidelberg, Germany; 4 National Koranyi Institute for Pulmonology, Budapest, Hungary; 5 Department of Medical Imaging, Radboud University Medical Center, Nijmegen, The Netherlands; 6 Department of Medicine and Surgery, University of Parma, Parma, Italy; 7 Fondazione IRCCS Istituto Nazionale dei Tumori of Milan, Milan, Italy; 8 Department of Genetics and Clinical Immunology, National Institute of Tuberculosis and Lung Diseases, Warsaw, Poland; 9 Department of Neurosurgery, University Hospital Mannheim, University of Heidelberg, Mannheim, Germany; 10 Department of Biomedical Imaging and Image-Guided Therapy, Medical University of Vienna, Vienna, Austria

**Keywords:** Lung cancer screening, Workload, Low-dose computed tomography, SOLACE, Radiology

## Abstract

**Objectives:**

Lung cancer screening (LCS) with low-dose computed tomography (LDCT) significantly reduces mortality among high-risk individuals, yet implementation remains limited at national and European levels. The Strengthening the Screening of Lung Cancer (SOLACE) Project aims to facilitate the implementation of equitable LCS. While considering the overall impact of LCS, we identified a lack of systematic evaluation on the impact on the workload of radiology professionals, including radiologists and radiographers. This multicentric, European-wide study prospectively examines the effects of LCS on radiology healthcare workers.

**Materials and methods:**

A survey-based cross-sectional study was conducted among European radiology professionals engaged in LCS to evaluate their experiences across seven domains: impacts on workload, workflow specifics, reporting practices, artificial intelligence (AI) use, participation in training programmes, potential professional development, and overall support for LCS.

**Results:**

We evaluated 56 responses (mean age 46 ± 10.8 years; 27 men) from 14 countries. About half of respondents (53.6%; *n* = 30) reported increased working hours, but only 21.6% (*n* = 11) felt overworked due to LCS. No significant factors were directly associated with feeling overworked. Most respondents (86.7%; *n* = 39) perceived increased expertise in assessing LCS LDCTs throughout screening, and 91.1% (*n* = 51) expressed support for the implementation of LCS.

**Conclusion:**

Although LCS increased workload for many radiology professionals, few reported feeling overworked. Despite the added demands, there may be added benefits for radiology professionals. Taken together, these findings emphasise the importance of continuously evaluating and monitoring how LCS affects the workload of radiology healthcare professionals.

**Key Points:**

***Question*** As wider implementation of lung cancer screening occurs in Europe, it is critical to consider its impact on radiology professionals’ workload and associated workforce demands.

***Findings*** Although lung cancer screening increased workload for many radiology professionals, few felt overworked, and the majority perceived increased diagnostic confidence and support for screening implementation.

***Critical relevance statement*:**

Lung cancer screening via LDCT plays a critical role in detecting cancer earlier and improving survival. With growing support for the broader implementation of LCS, it is essential to consider its potential impact on the workload of radiology professionals.

**Graphical Abstract:**

**Figure:**
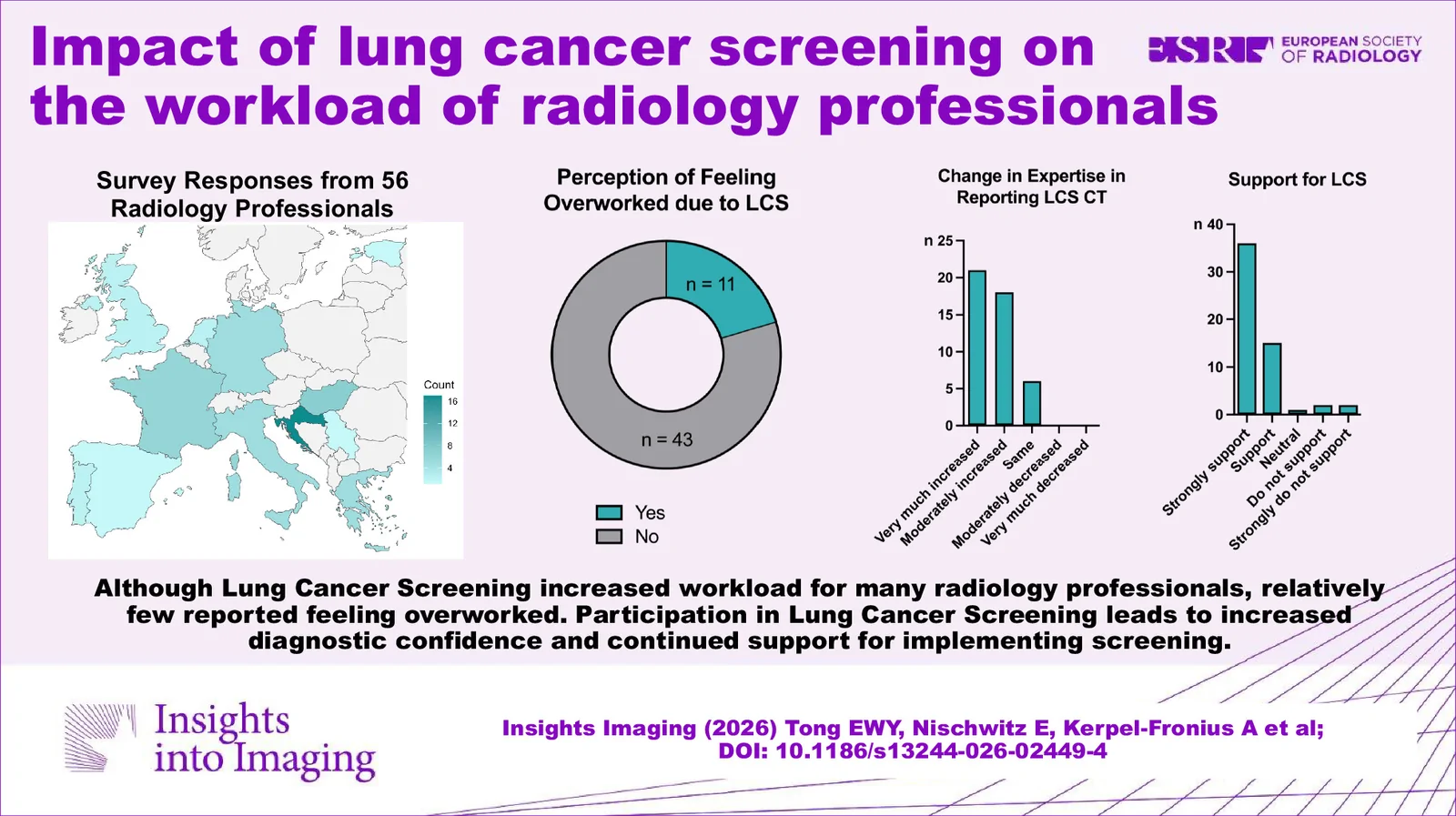

## Introduction

Lung cancer is the most diagnosed cancer worldwide. Global cancer statistics reported 2.5 million new cases worldwide in 2022, representing 12.4% of all cancer diagnoses [1]. Moreover, lung cancer is the leading cause of cancer-related death, accounting for over 1.8 million deaths in 2022 [1]. With lung cancer incidence projected to rise, driven by persistent smoking habits, ageing populations, and increasing air pollution, early detection is increasingly important [2]. This underscores the significant global health burden of lung cancer and highlights the critical need for effective prevention, early detection, and treatment strategies [3]. Lung cancer screening (LCS) via low-dose computed tomography (LDCT) has been shown to reduce lung cancer–specific mortality by 20–24% in high-risk populations by detecting lung cancer in asymptomatic individuals at earlier stages, as demonstrated in landmark trials such as the National Lung Screening Trial (NLST) [4] and NEderlands-Leuvens Longkanker Screenings ONderzoek (NELSON) [5]. Consequently, LCS programmes are being increasingly adopted globally, including recent policy endorsements across Europe [6, 7].

While the benefits of LCS are well-established, there is limited understanding of the quantitative workload implications on healthcare infrastructure, particularly in diagnostic imaging. Radiology departments are under increasing strain due to a confluence of three main factors: the rising global imaging market by 5.1% annually [8], increasing amount of cross-sectional imaging by 3–7% annually [9], and persistent workforce shortages [10, 11]. Hung et al confirmed a 20-fold increase in LDCT screening within a 13-year period (from 245 in 2008 to 4912 in 2017) [12]. In addition, LCS can intensify the workload on all radiology professionals, including radiologists and radiographers. For example, recent estimates in the United Kingdom suggest that full implementation of a national programme would require a substantial increase in staffing requirements, including an additional 75 radiologists and 184 radiographers [13]. A recent study reported that 76.7% of German radiologists already experience burnout, with increased workload and time pressure identified as significant contributing factors [14]. These findings emphasise the urgent need to assess and address the impact of LCS on radiology workload and professional well-being.

As LCS continues to expand, understanding its operational consequences is essential to ensure both effectiveness and system resilience. Hence, a Europe-wide survey-based cross-sectional study was conducted targeting LCS radiology professionals. Parameters such as the number of LDCT examinations, reporting duration, and distribution of procedural responsibilities were collected, alongside the subjective perception of being overwhelmed, programme satisfaction, and perceived diagnostic confidence. Additional emphasis was placed on the use of artificial intelligence (AI) and the role of LCS training as possible avenues for support. Through this approach, evidence-based insights were generated to guide sustainable LCS programmes that support healthcare workers.

## Materials and methods

This prospective, cross-sectional survey was conducted among European radiology healthcare professionals involved in LCS. The study protocol was conducted in accordance with the Declaration of Helsinki (2013). Participation was voluntary, anonymous, and uncompensated. Informed consent was given by all participants. Full compliance with applicable data protection regulations was ensured. The survey was developed by a radiologist with over 8 years of research experience and was reviewed by a multidisciplinary panel of LCS experts representing all SOLACE partners. Final approval was granted by SOLACE leadership. The survey was distributed to radiology professionals involved in LCS through the SOLACE consortium, the European Society of Thoracic Imaging (ESTI), and LCS sites involved in the NELSON trial, HANSE trial, 4-in-the-Lung-RUN trial, Croatian/Czech national programmes, and during the 2025 European Congress of Radiology. Responses were collected from April 2024 to March 2025.

### Survey

The survey comprised 50 questions in seven sections and was implemented using Google Forms (Supplementary Fig. 1). Survey respondents were initially screened to ensure only radiology professionals participated. Partial responses, defined as failing to meet the minimum completion threshold (see Supplementary Fig. 1), were excluded from final analysis. A total of 60 participants responded to the survey, of which 56 participants responded to all essential questions. These responses were included in the final analysis. The first section included questions about respondents’ demographics. The second section comprised questions on workload, such as the number of weekly working hours and CT examinations (routine examinations and LCS LDCT examinations), and how and why this changed due to the implementation of LCS. Respondents described which steps they contributed to in LCS and the duration spent. Finally, respondents were asked if they personally feel overworked by LCS.

The third section consisted of questions regarding reporting LCS LDCT, such as reporting format, time required to generate a report, and how this differs from routine chest CT examinations. The fourth section explored whether respondents regularly used AI and which AI software they utilised. Respondents indicated how supported they feel by these software programs and if their workload and confidence levels changed in response to LCS. In the fifth section, respondents were asked to specify if they attended a dedicated LCS training programme and to provide details on the programme, if applicable. The sixth section focused on the potential benefits of LCS on radiologists’ expertise, confidence and time taken for an LCS report. In the seventh and final section, respondents expressed the extent to which they personally support LCS and their satisfaction with LCS in their institution and country.

The survey had varying question types, including binary questions, rating answers based on a Likert scale, free text fields, and multiple-choice questions. The full questionnaire is found in Supplementary Fig. 1.

### Statistical analysis

Categorical and continuous variables were summarised with descriptive statistics, including frequency distribution, mean, and median. The association between a categorical dependent variable and two or more independent groups was evaluated using Fisher’s exact test. To analyse the association between a categorical dependent variable and multiple independent variables, a generalised linear model was used. To measure if various factors relating to workload were related to one another, Spearman’s correlation was employed. To compare the demographics and survey responses between radiologists and radiographers, Fisher’s exact test and Mann–Whitney test were applied for categorical and continuous variables, respectively. A *p*-value less than 0.05 was considered significant. Where possible, the hazard ratio and 95% confidence interval were calculated. Analysis was carried out via R 4.4.2 (The R Foundation, Vienna, Austria) and GraphPad Prism version 10.4.1. (GraphPad software, Boston, MA, USA, www.graphpad.com).

## Results

### Participant demographics

**Fig. 1:**
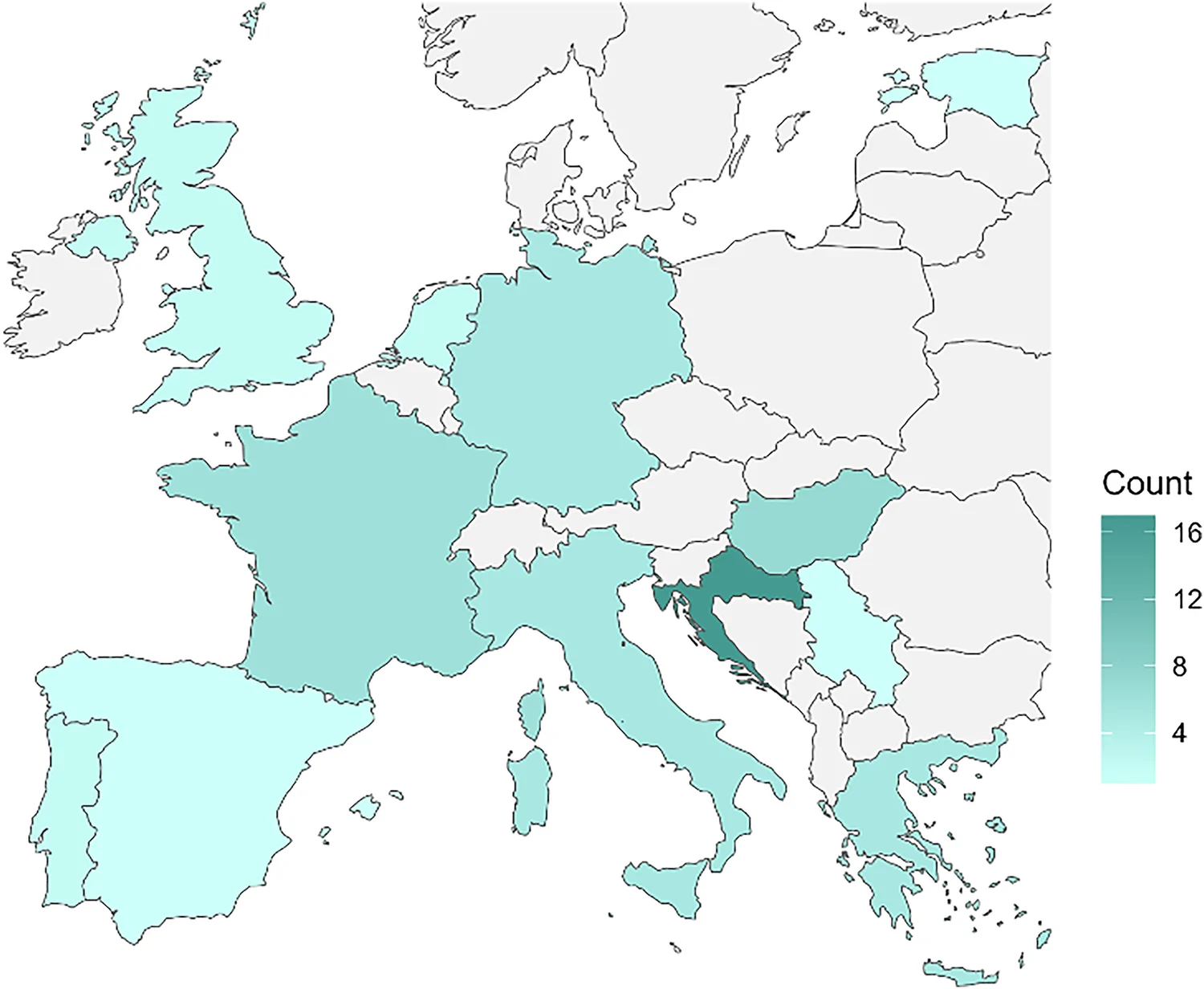
Overview of survey responses by country. Countries are shaded in teal according to the distribution of responses, with the lighter shade indicating fewer responses (*n* = 1) and the darker shade indicating more responses (up to *n* = 17). Countries with no response are shown in grey (*n* = 55)

Responses from 56 individuals (mean age, 46 ± 10.8, 27 men) from 14 different European countries were included in the final analysis (Table 1 and Fig. 1). Respondents had a median of 19 years (± 11.6) of medical-related working experience. The study involved 40 radiologists (71.4%), 14 radiographers (25%) and two from other screening-related jobs (3.6%), including a screening centre assistant and a technical physician. If there were any significant deviations between the responses provided by these groups (Supplementary Table 1), it has been explicitly noted in the text. Among all respondents, the majority worked in an academic hospital (*n* = 31; 55.3%), followed by an approximately even distribution in private practice (*n* = 8; 14.2%), dedicated lung screening centre or research centre (*n* = 8; 14.2%), and non-academic hospitals (*n* = 9; 16.1%). Additionally, the respondents represent an equal distribution of population-based screening programmes, regional programmes, and implementation pilots. Most individuals had either a subspeciality in chest radiology (23, 41.1%) or no subspeciality (*n* = 21; 37.5%) (Table 1).

**Table 1:** Descriptive statistics of survey participants

Participants	56
Age, years	46 (± 10.8)
Medical experience, years	19 (± 11.6)
Sex	
Male	27 (51.8%)
Female	29 (48.2%)
Occupation	
Radiologist	40 (71.4%)
Radiographer	14 (25.0%)
Other	2 (3.6%)
Country	
Croatia	17 (30.4%)
Hungary	7 (12.5%)
France	6 (10.1%)
Greece	5 (8.9%)
Germany	5 (8.9%)
Italy	5 (8.9%)
Other	10 (17.9%)
Primary practice setting	
Academic hospital	31 (55.3%)
Non-academic hospital	9 (16.1%)
Dedicated lung screening/research centre	8 (14.2%)
Private practice	8 (14.2%)
Primary subspecialty	
Chest	23 (41.1%)
None	21 (37.5%)
Other	12 (21.4%)

### Effects of the implementation of LCS on workload

**Fig. 2:**
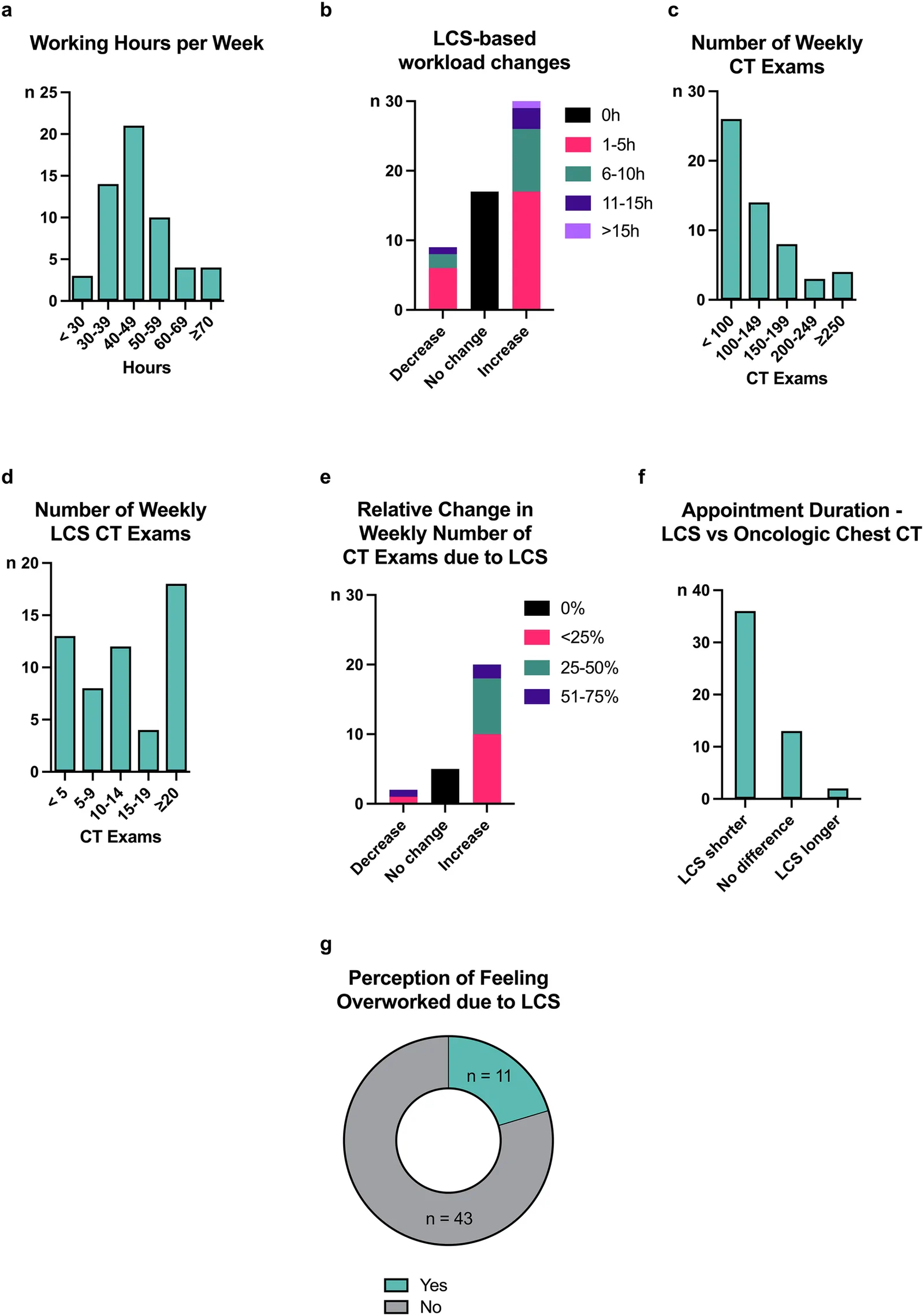
Effects of the implementation of LCS on the workload of radiology staff. **a** Average weekly working hours reported by participants. **b** Perceived change in workload due to lung cancer screening. Hours are categorised as 0 h (black), 1–5 h (pink), 6–10 h (turquoise), 11–15 h (dark purple), > 15 h (light purple). **c**, **d** Reported average number of CT examinations performed per week overall (**c**) and specifically for LCS (**d**). **e** Reported percentage change in the number of CT exams performed due to LCS, categorised as 0% (black), < 25% (pink), 25–50% (turquoise), and 51–75% (dark purple). **f** Comparison between the time required between a LCS CT and an oncologic chest CT examination. Respondents indicated if the appointment duration for an LCS CT was shorter, equal, or longer than an oncologic chest CT. **g** Pie chart depicting the proportion of participants who reported feeling overworked due to LCS. For **a**–**g**, bar plots and stacked bar plots show the number of respondents (*n*) on the *y*-axis

Most respondents (*n* = 39; 69.6%) reported working 40 h or more per week (Fig. 2a). Overall, 53.6% (*n* = 30) indicated that LCS contributed to an increase in working hours, with the most frequently reported increment being 1–5 h per week (*n* = 17; 30.3%) (Fig. 2b). Most frequently (*n* = 29, 52.7%), respondents reported conducting more than 100 CT examinations per week. Approximately one-third (*n* = 18) of respondents reported performing more than 20 chest LCS LDCT per week (Fig. 2d). Most participants (*n* = 38; 71.7%) stated that implementing LCS has increased their weekly number of CT examinations, with the largest proportion (*n* = 10; 37%) indicating an increase by up to 25% (Fig. 2e). When asked for reasons for this increase, 18 out of 20 (90%) respondents described the additional LDCT as the contributing factor. There was also a significant positive correlation between an increase in working hours and the number of LCS LDCT examinations (r = 0.29, *p*-value = 0.032) (Supplementary Fig. 2a). To further probe the impacts of LCS on the workload of radiology professionals, respondents were asked to describe the workflow in LCS appointments. The most frequently reported time interval, both scheduled by the screening centre and required to complete all LCS steps, was 6–10min. The amount of time required to complete all LCS steps negatively correlated with the number of working hours (correlation coefficient r = −0.41, *p*-value = 0.004) and the number of CT examinations per week (correlation coefficient = −0.4, *p*-value = 0.004) (Supplementary Fig. 2a). A timeslot for LCS was reported by most respondents (*n* = 36, 73.5%) to be shorter than an oncologic chest CT examination, with the most frequent cause cited as the lack of need for contrast agents (Fig. 2f and Supplementary Fig. 2b).

The minority of respondents (*n* = 11, 20.4%) indicated that they felt personally overworked due to the addition of LCS to their workload (Fig. 2g). No significant variables influencing the perception of feeling overworked were found (Supplementary Fig. 2c).

### Influence of the use of structured reporting, AI and LCS training programmes on reporting LDCTs

**Fig. 3:**
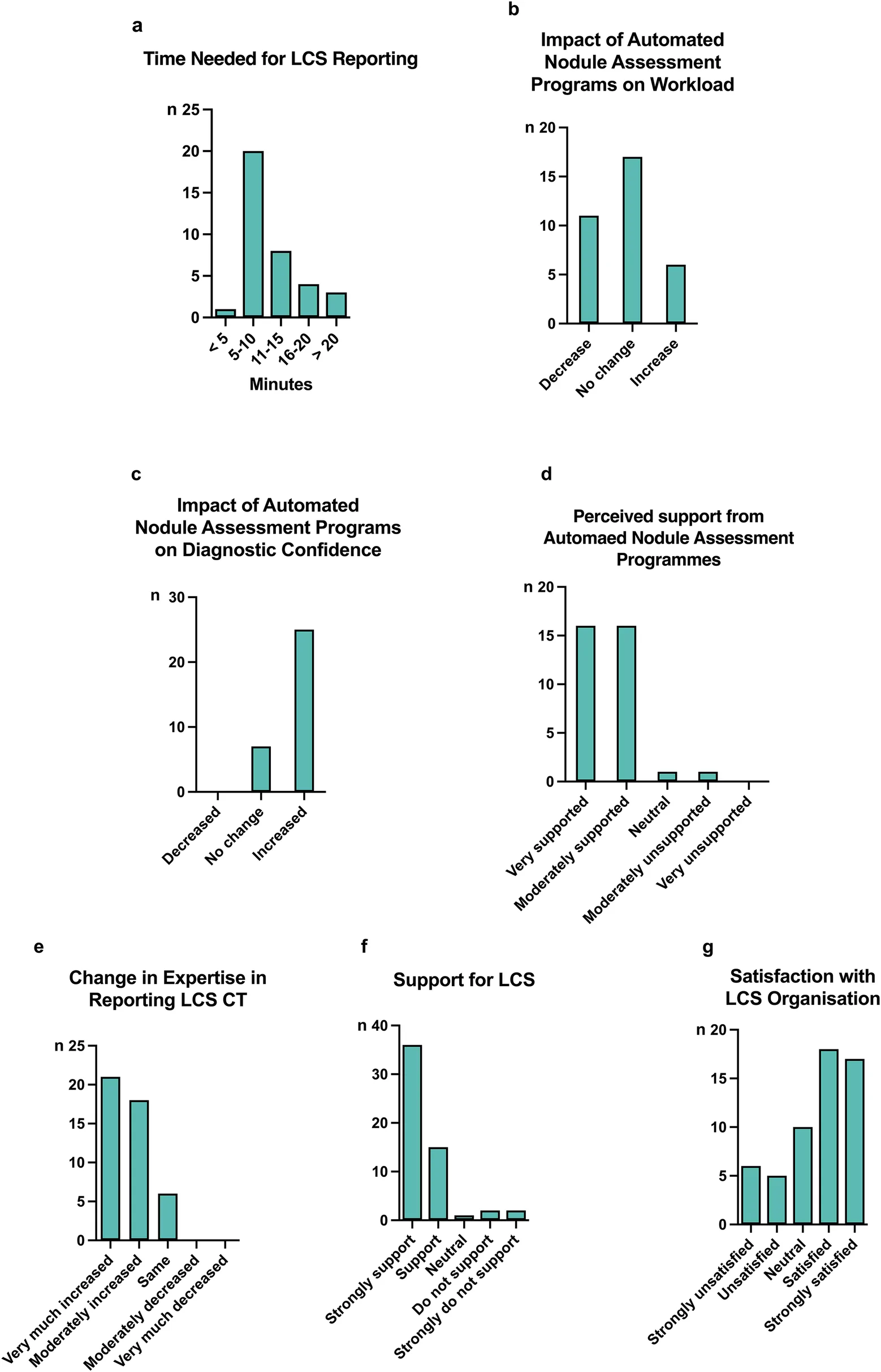
Effects of automated nodule assessment programmes on LCS reporting and influences of LCS on radiologist professionals’ expertise and overall support. **a** The reported average time (minutes) needed for each LCS report. **b**–**d** Reported impact on workload (**b**), the effect on diagnostic confidence (**c**), and the perceived support (**d**) from AI assistance. **e** Perceived change in level of expertise in reporting LCS CT examinations. **f**, **g** Overall support of LCS (**f**) and satisfaction with its organisation (**g**). For all panels, bar plots show the number of respondents (*n*) on the *y*-axis

Imaging reporting represents a notable aspect of radiologists’ workload. The majority of respondents (*n* = 28; 77.8%) estimated spending between 5 and 15 min to complete a LCS report, and most (*n* = 29; 82.9%) used structured reports (Fig. 3a). Compared to oncologic chest CT examinations, the time required for LCS LDCT reporting was perceived as shorter by 47.2% (*n* = 17) of respondents, while 36.1% (*n* = 13) indicated it was approximately the same (Supplementary Fig. 3a).

To further explore aspects of LCS reporting, respondents were asked about the use of, and experiences with, AI tools in this context. The majority of the respondents (*n* = 39; 69.6%) reported using automated nodule assessment programs in clinical practice, with 92.3% (*n* = 36) of these incorporating them into LCS workflows. Opinions on the impact of automated analysis programs on individual workload varied among respondents. A total of 50.0% (*n* = 17) of respondents reported no change in workload, while 32.4% (*n* = 11) noted a reduction and 17.7% (*n* = 6) reported an increase (Fig. 3b). Nevertheless, most users (*n* = 25; 86.2%) indicated that automated nodule assessment tools enhanced their precieved diagnostic confidence in decision-making (Fig. 3b) and further 94.1% (*n* = 32) of respondents felt moderately or very supported by these tools (Fig. 3d).

In addition to AI tools, radiologists can be supported by LCS-targeted training. Slightly more than half of the respondents (56.6%, *n* = 30) reported having dedicated LCS training. Access to LCS-targeted training was also positively associated with increased diagnostic confidence in LCS reporting (odds ratio 1.24, 95% CI: 1.01–1.52; *p* = 0.04) (Supplementary Fig. 3b).

Altogether, 86.7% (*n* = 39) of respondents indicated a moderately or very much increased reporting expertise in chest CT throughout LCS (Fig. 3e).

### Satisfaction of professionals with LCS programme

The majority of respondents support or strongly support the implementation of LCS (*n* = 51; 91.1%) (Fig. 3f). Satisfaction with the local organisation of LCS was reported by 62.5% (*n* = 35) of the respondents, whereas 19.6% (*n* = 11) were unsatisfied (Fig. 3g). Of note, this was the only question in which the distribution of responses between radiologists and other radiology-related personnel were significantly different (*p* = 0.05). The responses from radiologists to this question were approximately split, with 52.5% (*n* = 21) reporting satisfaction and 47.5% reporting dissatisfaction, whereas the majority of radiographers and other indicated professions (*n* = 12, 85.7%) reported satisfaction. The level of satisfaction with the organisation of LCS in participants’ institutions did not have a significant association with the feeling of being overworked in either group (Supplementary Fig. 2c).

## Discussion

This prospective, survey-based study is the first to explore the impact of LCS on radiology professionals’ workload. The majority of respondents (71.0%, *n* = 38) reported an increase in CT numbers, and 53.6% (*n* = 30) indicated that LCS had led to longer working hours. However, only 21.6% (*n* = 11) of respondents reported feeling overworked, suggesting that LCS-related demands do not necessarily translate into perceived overload. Notably, 86.7% (*n* = 39) of respondents perceived an improvement in LCS reporting expertise, likely due to focused chest reporting. Beneficial effects were also reported from both LCS training and automated nodule assessment tools. Dedicated certification and training were positively associated with reported diagnostic confidence (OR 1.24, 95% CI: 1.01–1.52; *p* = 0.04). Automated imaging analysis tools were also well received, with 86.2% (*n* = 25) indicating improved diagnostic confidence and 94.1% (*n* = 32) feeling supported by their use.

### Effects of the implementation of LCS on workload

Even without the increased workload associated with LCS, recent studies have highlighted the overall increase in workload among radiology staff. Bruls and Kwee found that the workload of radiologists during on-call shifts experienced a marked increase in the last 15 years, attributed mainly to the increased number of CT examinations [15]. Furthermore, radiologists are expected to carry out non-medical tasks, such as administrative work, teaching, and research, often outside of traditional working hours [16]. Having personal contact with both screening participants and patients, radiographers must work efficiently and effectively, while upholding service standards [17]. The implementation of LCS is expected to increase the workload in radiology, as LCS examinations are likely to increase follow-up diagnostics, and the staff of radiology departments is not expected to be extended accordingly. The results of this survey support this assumption, demonstrating a positive correlation between the number of CT examinations performed for LCS and an increase in working hours (r = 0.29, *p*-value = 0.032). The number of working hours and the number of CT examinations per week were both inversely correlated (r = −0.40, *p* = 0.004) with the time required to complete the LCS workflow per participant. This suggests that in settings with higher workload, the screening process may be carried out more efficiently, likely because greater participant volume limits the amount of time healthcare workers can dedicate to each individual. Another contributing factor to the increased workload is that the LCS LDCT timeslot is shorter than for a standard oncologic chest CT, leading to a higher turnover within the same timeframe. Moreover, as LCS participants are expected to be in better health and asymptomatic, they tend to be more cooperative and independent, improving workflow processes [18, 19].

While 53.6% (*n* = 30) of respondents reported that LCS increased their working hours, only 21.6% (*n* = 11) indicated feeling overworked. This proportion is notably below previously reported rates of burnout of 77% and 79% among radiologists in Germany [14] and the United States [16], respectively. Furthermore, no variables were found to be directly associated with the perception of feeling overworked. This suggests that an increased workload from LCS does not inherently result in heightened stress or fatigue among respondents, possibly due to the relatively lower complexity and higher standardisation of LCS LDCT interpretation. LCS exams are low-dose, non-contrast scans focused on pulmonary nodule detection, often reported using structured systems such as Lung-RADS, which are designed to streamline workflow and reduce reporting variability [20]. This may explain why the increased exam volume did not translate into a higher perception of overwork. Additionally, with baseline nodule detection rates in screening populations reported at 11.4–17.7% [21], most LCS exams are not found to have suspicious nodules. This may simplify Lung-RADS classification, which may further contribute to a more streamlined and standardised reporting process. Correspondingly, 47.2% (*n* = 17) of respondents indicated that the time taken to report a LCS scan was shorter than a normal oncologic chest CT examination.

Another important consideration is the potential for bias in the survey respondent pool. It is likely that the individuals who were targeted and chose to respond are at the forefront of LCS and therefore support its implementation, which may have influenced them to answer more positively. This interpretation is reinforced by the fact that the vast majority of the respondents expressed support or strong support for LCS implementation (*n* = 51; 91.1%). These findings highlight the importance of continual evaluation of the impact of LCS on the workload of healthcare professionals throughout the implementation of LCS.

### Impact of structured reporting, AI, and LCS training on image assessment and workload

Writing radiology reports constitutes a significant proportion of radiologists’ working hours. In our study, LCS structured reporting is used by 82.9% (*n* = 29) of survey respondents, aligning with recommendations from the European Society of Radiology [7] and the American College of Radiology, as standardised terminology enhances clarity and communication for both physicians and LCS participants [22]. As imaging findings in LCS tend to be less frequent and complex than in diagnostic imaging, LCS reporting may align well within predefined templates. Hence, structured reporting in LCS may offer a more efficient and convenient approach [23]. Emphasising the advantages of structured reporting, a study by Cereser et al demonstrated improved report quality in lung cancer staging CT examinations compared to narrative reporting [24].

The use of automated nodule assessment programs in LCS was reported by 65.5% (*n* = 36) of the participants. Approximately half of the survey respondents (50%; *n* = 17) reported AI did not lead to workload change, and only 32.4% (*n* = 11) noted a workload reduction. There are, to the best of our knowledge, no studies to date which report on the effect of AI on workload change in LCS, although there are studies that investigated the effect that AI has on reading time in reader studies [25–27]. AI as a first-reader “rule-out” filter in LCS LDCT achieved a negative predictive value (NPV) of 99.8–99.9% and was estimated to support reducing reading workload by 74.7–79% by excluding the majority of negative examinations from full radiologist review [28, 29]. Consistently, implementation analyses in screening cohorts showed that commercial AI tools can accurately flag negative LDCTs, supporting reductions in radiologist review workload [30]. A simulated AI prescreener strategy reduced mean interpretation time from 164 s to 143 s and improved specificity from 88.8% to 90.3% without the loss of sensitivity [31]. A systematic review of CE-marked AI software including 11 studies (19,770 participants) reported sensitivity increases of 5–20% accompanied by specificity decreases of 3–7% [32]. In 4-IN-THE-LUNG-RUN baseline screening (*n* = 3678), AI reduced negative misclassifications compared to radiologists (0.8% vs 11.1%) and lowered missed clinical referrals (2.9% vs 11.8%). These findings are consistent with safer triage and fewer downstream re-reads, resulting in a more efficient allocation of radiologist workload [33].

These findings align with the perceptions observed in the present study, in which 32.4% of respondents reported a reduction in workload, while 86.2% indicated increased diagnostic confidence and 94.1% felt supported in clinical decision-making by digital and AI-based tools. This suggests that such tools may contribute to improved workflow efficiency while strengthening diagnostic certainty. Furthermore, most respondents (86.7%, *n* = 39) perceived increased expertise in reporting LCS LDCT and evaluating lung nodules throughout the screening, perhaps due to increased amounts of focused chest CT reporting [34]. Participation in LCS training programmes was associated with perceived increased diagnostic confidence, likely reflecting improved competence acquired through structured educational interventions [35]. Although the extent to which training and focused experience in LCS improves reporting quality and reduces missed diagnoses has not been formally quantified, evidence from breast cancer screening suggests a significant benefit. Geertse et al retrospectively examined 18 programmes that incorporated supplementary training, with 17 showing improved outcome measures as a result of this training [36].

### Limitations

This study is subject to several limitations. Most notably, it relied on a limited sample size of radiology professionals actively involved in LCS programmes, given that there are currently a limited number of LCS programmes in Europe. In addition, for ease of completion, not all questions were designated as mandatory. To ensure transparency and an accurate representation of the findings, we have included the raw number of responses for each question discussed. Moreover, all data were based on subjective self-reports, with no control group or objective workload metrics (e.g., actual CT volumes or time-based workflow analysis), limiting the ability to quantitatively validate perceived workload changes. There is still a large need to evaluate changes in workload with objective workload measurements.

While the Europe-wide scope of the survey is one of the primary strengths of the studies, it is critical to consider that the responses reflect the heterogeneous geographic and sociodemographic factors which influence the distribution and implementation of LCS services. A population-based study showed that 5% of the eligible U.S. population had no access to screening facilities within 40 miles, with substantially lower access in rural regions [37]. Consistently, screening utilisation remains low overall, with in the U.S. only 4.1% (95% CI, 3.9–4.3%) of eligible Medicare beneficiaries (*N* = 103,892) undergoing LDCT and significant regional and demographic variation, specifically with lower screening frequency in non-white individuals (*p* < 0.001) and geographically highest screening rate in the Northeast [38]. In a cohort of 1268 eligible individuals, LDCT uptake did not differ between rural and urban populations (16.3% vs 17.7%; OR = 0.85, 95% CI: 0.67–1.09) but was lower among those reporting financial barriers or lacking primary care access, and higher in those with chronic comorbidity burden and poor overall health status [39]. These findings indicate that geographic access, socioeconomic conditions, and population health characteristics influence screening utilisation and may result in unequal distribution of screening-related workload.

Reimbursement-linked requirements, including mandatory shared decision-making consultations, introduce additional organisational steps and documentation demands that extend beyond image interpretation and increase the overall workload associated with screening delivery [40]. In addition, population-level implementation of LDCT screening has been projected to increase imaging volumes by approximately 4% overall, with substantially higher increases in socioeconomically disadvantaged and underserved regions, further contributing to regional concentration of screening workload [41]. Taken together, these structural and reimbursement factors indicate an unequal distribution and potential concentration of screening-related clinical effort.

Given the heterogeneous socioeconomic conditions, geographical differences, as well as varying healthcare and reimbursement structures across European LCS programmes in this study, a comparative analysis of these effects on radiology-associated workload was not feasible. Further Europe-wide analyses are required to evaluate the impact of structural and socioeconomic variation on screening workload and implementation.

Additionally, we highlight the likely selection bias with the respondents, which likely reflects radiology professionals at the forefront of LCS and therefore supports its implementation, which may have influenced them to answer more positively than the overall population. Moreover, findings may have been influenced by the relatively high proportion of respondents from Croatia. This overrepresentation may limit the generalisability of the findings to countries without established national screening programmes. Country-specific healthcare structures and screening implementation may have shaped participants’ experiences and perceptions.

## Conclusion

While respondents reported increased CT volumes and extended working hours caused by LCS, most respondents did not perceive a corresponding increase in workload burden. The reported improvement in diagnostic expertise, possibly attributable to focused chest CT reporting, suggests a positive learning effect through routine exposure. Furthermore, participation in structured LCS training and the use of automated nodule assessment tools were both significantly associated with perceived increased diagnostic confidence, underscoring their potential to support radiologists in maintaining accuracy and efficiency in LCS workflows. Taken together, these findings emphasise the importance of continuously evaluating and monitoring how LCS affects the workload of radiology healthcare professionals.

## Supplementary information

ELECTRONIC SUPPLEMENTARY MATERIAL

## Data Availability

The datasets generated during the current study are available from the corresponding author upon reasonable request. Access may be subject to institutional policies, ethical approval, and data-sharing agreements.

## Abbreviations

AI: Artificial intelligence
CT: Computed tomography
LCS: Lung cancer screening
LDCT: Low-dose computed tomography
NELSON: Nederlands-Leuvens Longkanker Screenings ONderzoek
SOLACE: Strengthening the screening of lung cancer
