# Supplementary material for: Impact of lung cancer screening on the workload of radiology professionals
Source: Insights Imaging. 2026 Nov 12;17:293. doi: 10.1186/s13244-026-02449-4 (PMC13759930; doi:10.1186/s13244-026-02449-4)
Supplement: Supplementary file 1 [file 13244_2026_2449_MOESM1_ESM.pdf]

Impact of lung cancer screening on the workload of radiology professionals

ELECTRONIC SUPPLEMENTARY MATERIAL

Insights Imaging (2026) Tong EWY, Nischwitz E, Kerpel-Fronius A et al.

Supplementary Figures

Supplementary Figure 1: Outline of "Opinions Concerning Lung Cancer Screening Survey".

Overview

1. Is your profession directly related to the field of radiology?*
   a. Yes -> "Demographics" section
   b. No -> Thank you for your interest in supporting the SOLACE project! However, at this time we are currently looking for feedback from radiology-associated personnel. If you are interested in learning more about the SOLACE project, please visit our website: https://europeanlung.org/solace/

Demographics

2. Gender*
   a. Female
   b. Male
   c. Other (FT)

3. Age (FT)*

4. Which screening centre are you associated with?*
   a. Croatia
   b. Czechia
   c. Estonia
   d. France-APHP
   e. France-HCL
   f. Germany-DKFZ
   g. Germany-GHD/MHH/UKSH
   h. Greece
   i. Hungary
   j. Ireland
   k. Italy-INT-IRCCS
   l. Italy-UNIPR
   m. Poland
   n. Spain
   o. Other (FT)

5. Country of your institution:*
   a. Austria
   b. Belgium
   c. Bulgaria
   d. Croatia
   e. Republic of Cyprus
   f. Czech Republic
   g. Denmark
   h. Estonia
   i. Finland
   j. France
   k. Germany
   l. Greece
   m. Hungary
   n. Ireland
   o. Italy
   p. Latvia
   q. Lithuania
   r. Luxembourg
   s. Malta
   t. Netherlands
   u. Poland
   v. Portugal
   w. Romania
   x. Slovakia
   y. Slovenia
   z. Spain
   aa. Sweden
   bb. Other (FT)

6. Working position that best describes your current role:*
   a. Radiographer
   b. Screening Centre Nurse
   c. Screening Centre Assistant
   d. Radiologist
   e. Other (FT)

7. If you are a radiologist, indicate specific position:*
   a. I am not a radiologist.
   b. Resident (in training)
   c. Associate Specialist/Fellow
   d. Consultant/Attending Physician
   e. Leading Position/Head of the department

8. Years of medical-related working experience: (FT)*

9. Primary subspecialty:*
   a. None
   b. Emergency radiology
   c. Musculoskeletal radiology
   e. Oncologic imaging
   f. Paediatric Radiology
   g. Thoracic Radiology

d. Neuroradiology
h. Other (FT)

10. Primary practice setting:
    a. Dedicated Lung Cancer Screening Centre
    b. Academic Hospital
    c. Non-Academic Hospital
    d. Private Practice
    e. Other (FT)

Workload

11. Current working hours per week:
    a. < 30 hours
    b. 30-39 hours
    c. 40-49 hours
    d. 50-59 hours
    e. 60-69 hours
    f. ≥70 hours

12. Number of total CT examinations that you perform per week:
    a. < 100 CTs
    b. 100-149 CTs
    c. 150-199 CTs
    d. 200-249 CTs
    e. ≥250 CTs

13. Number of chest CTs only for lung cancer screening that you perform per week:
    a. < 5 LCS CTs
    b. 5-9 LCS CTs
    c. 10-14 LCS CTs
    d. 15-19 LCS CTs
    e. ≥20 LCS CTs

14. Indicate the amount your workload decreased or increased due to lung cancer screening:*
    a. 0 hours-no change
    b. Decrease by 1-5 hours
    c. Decrease by 6-10 hours
    d. Decrease by 11-15 hours
    e. Decrease >15 hours
    f. Increase by 1-5 hours
    g. Increase by 6-10 hours
    h. Increase by 11-15 hours
    i. Increase by >15 hours

15. If an increase or decrease in workload occurred, what may be reasons for this change? (FT)

16. Do you personally feel overworked because of lung cancer screening?
    a. Yes
    b. No

17. Do you perform lung cancer screening exclusively?
    a. Yes
    b. No

18. Do you perform lung cancer screening during clinical/diagnostic routine?
    a. Yes
    b. No

19. Did the total number of CT examinations change because of lung cancer screening*
    a. Increase
    b. Equal
    c. Decrease

20. System booked duration of the CT time slot for lung cancer screening in your department:
    a. < 5 minutes
    b. 5-10 minutes
    c. 11-15 minutes
    d. 16-20 minutes
    e. ≥20 minutes

21. Does the duration of a timeslot for lung cancer screening CT differ from the duration of an oncologic chest CT?
    a. Length of LCS > terminated oncologic chest CT slots.

b. Length of LCS = terminated oncologic chest CT slots.
c. Length of LCS < terminated oncologic chest CT slots.

22. What are potential reasons for this time difference? (FT)

23. Approximate amount of time spent to complete all necessary lung cancer screening steps:
a. ≤ 5 minutes
b. 6-10 minutes
c. 11-15 minutes
d. 16-20 minutes
e. ≥20 minutes

24. Which of these parts of lung cancer screening do you contribute to? (check all that apply)
a. Welcoming patient
b. General introduction/ explanations
c. Safety check
d. Conducting the CT exam
e. Helping the patient on and off the table
f. Post-examination disinfection
g. Post-exam check-up on the patient
h. Image reconstructions
i. Finding communication to the patient
j. Interdisciplinary finding communication/ discussion
k. Other (FT)

25. Do you contribute to reporting as part of lung cancer screening?*
a. Yes (-> "Reporting" section
b. No (-> "Automated Image Assessment – Yes/No" section)

Reporting

26. Reporting is usually done as:
a. Free text
b. Structure reporting
c. Other (FT)

27. How much time do you need for a lung cancer screening imaging report?
a. < 5 minutes
b. 5-10 minutes
c. 11-15 minutes
d. 16-20 minutes
e. ≥20 minutes

28. How does the reporting time for lung cancer screening differ from routine chest CT images?
a. Reporting of lung cancer screening < routine chest CT images
b. Reporting of lung cancer screening = routine chest CT images
c. Reporting of lung cancer screening > routine chest CT images

29. Did the approximate number of chest CT per week change because of lung cancer screening?
a. Yes
b. No

30. If the approximate number of chest CTs per week changes because of lung cancer screening (LCS), by what percentage did they increase or decrease?
a. LCS did not change the number of CTs per week
b. LCS increased the number of CTs per week by less than 25%
c. LCS increased the number of CTs per week by between 25-50%
d. LCS increased the number of CTs per week by between 51-75%
e. LCS increase the number of CTs per week by more than 75%
f. LCS decreased the number of CTs per week by less than 25%
g. LCS decreased the number of CTs per week by between 25-50%
h. LCS decreased the number of CTs per week by between 51-75%
i. LCS decreased the number of CTs per week by more than 75%

Automated Image Assessment – Yes/No

31. Do you use automated nodule assessment programs on a regular basis?*
    a. Yes (-> "Automated Image Assessment" section)
    b. No (-> "Lung Cancer Screening Training Curriculum – Yes/No" section)

Automated Image Assessment

32. Do you use automated nodule assessment programs on a regular basis?*
    a. Yes
    b. No

33. Are these automated nodule assessment programs used for lung cancer screening?*
    a. Yes
    b. No

34. Please specify which program(s) is being used: (FT)

35. How supported do you feel by these program(s)?
    a. Very supported by these programs
    b. Moderately supported by these programs
    c. Not unsupported or supported by these programs
    d. Moderately unsupported by these programs
    e. Very unsupported by these programs

36. Did automated nodule assessment programs change your individual workload?
    a. There was no change in my workload due to automated nodule assessment programs.
    b. There was an increase in my workload due to automated nodule assessment programs.
    c. There was a decrease in my workload due to automated nodule assessment programs.

37. Indicate an approximate amount of hours that your workload has changed due to automated nodule assessment programs (indicating 0 if no change occurred): (FT)

38. Did the analysis programs have an impact on the level of confidence you had in making a correct diagnosis?
    a. Increased confidence
    b. Did not affect confidence
    c. Decreased confidence

Lung Cancer Screening Training Curriculum – Yes/No

39. Did you attend a dedicated lung cancer screening training?
    a. Yes (-> "Lung Cancer Screening Training Curriculum" section)
    b. No (-> "Expertise and Diagnostic Confidence" section)

Lung Cancer Screening Training Curriculum

40. Indicate the format of the training:
    a. On-site course
    b. Training workshop
    c. Internal supervision/guidance
    d. External internship
    e. Other (FT)

41. Please indicate particular organization of training, if known (FT)

42. Is lung cancer screening training curriculum mandatory in your department?
   a. Yes
   b. No

43. Indicate all topics addressed during the lung cancer screening training curriculum: (check all that apply)

   a. Technical basics
   b. Dose management
   c. Image quality
   d. Execution of a chest CT
   e. (Iterative) reconstructions
   f. Image viewing
   g. Reporting
   h. Standardized/ structured reporting
   i. Chest diagnoses
   j. Nodule assessment
   k. Scoring systems (LungRads, Fleischner criteria, etc.)
   l. Program based diagnostic support (e.g. automated nodule detection and qualitative image assessment)
   m. Other (FT)

Expertise and Diagnostic Confidence

44. How confident are you about your radiological diagnosis in lung cancer screening (LCS)?
   a. Very confident about radiological diagnosis in LCS
   b. Moderately confident about radiological diagnosis in LCS
   c. Neither confident or unconfident about radiological diagnosis in LCS
   d. Moderately unconfident about radiological diagnosis in LCS
   e. Very unconfident about radiological diagnosis in LCS

45. In your opinion, did your expertise in reporting chest CTs and evaluating nodules improve throughout the LCS, caused by an increased number of chest CTs?
   a. Very much increased expertise in reporting chest CTs and evaluating nodules throughout LCS
   b. Moderately increased expertise in reporting chest CTs and evaluating nodules throughout LCS
   c. Neither increased or decreased expertise in reporting chest CTs and evaluating nodules throughout LCS
   d. Moderately decreased expertise in reporting chest CTs and evaluating nodules throughout LCS
   e. Very much decreased expertise in reporting chest CTs and evaluating nodules throughout LCS

46. Has your time for reporting chest CTs changed throughout the LCS?
   a. Decreased amount of time
   b. Same amount of time
   c. Increased amount of time

47. Indicate percentage change in reporting time throughout lung cancer screening:

   a. No change in reporting time throughout LCS
   b. Less than 25% increase in reporting time throughout LCS
   c. 25-50% increase in reporting time throughout LCS
   d. 51-75% increase in reporting time throughout LCS
   e. More than 75% increase in reporting time throughout LCS
   f. Less than 25% decrease in reporting time throughout LCS
   g. 25-50% decrease in reporting time throughout LCS
   h. 51-75% decrease in reporting time throughout LCS
   i. More than 75% decrease in reporting time throughout LCS

Personal Opinions on Lung Cancer Screening

48. Do you personally support the implementation of lung cancer screening (LCS)?
    a. Strongly do not support the implementation of LCS
    b. Do not support the implementation of LCS
    c. Do not support or not support the implementation of LCS
    d. Support the implementation of LCS
    e. Strongly support the implementation of LCS

49. How satisfied are you with the organisation of lung cancer screening within your clinic/country?
    a. Strongly unsatisfied with the implementation of LCS within your clinic/country
    b. Unsatisfied with the implementation of LCS within your clinic/country
    c. Neither satisfied or unsatisfied with the implementation of LCS within your clinic/country
    d. Satisfied with the implementation of LCS within your clinic/country
    e. Strongly satisfied with the implementation of LCS within your clinic/country

*Indicates required questions.
Unless otherwise indicated, questions were multiple choice.

FT = Free Text

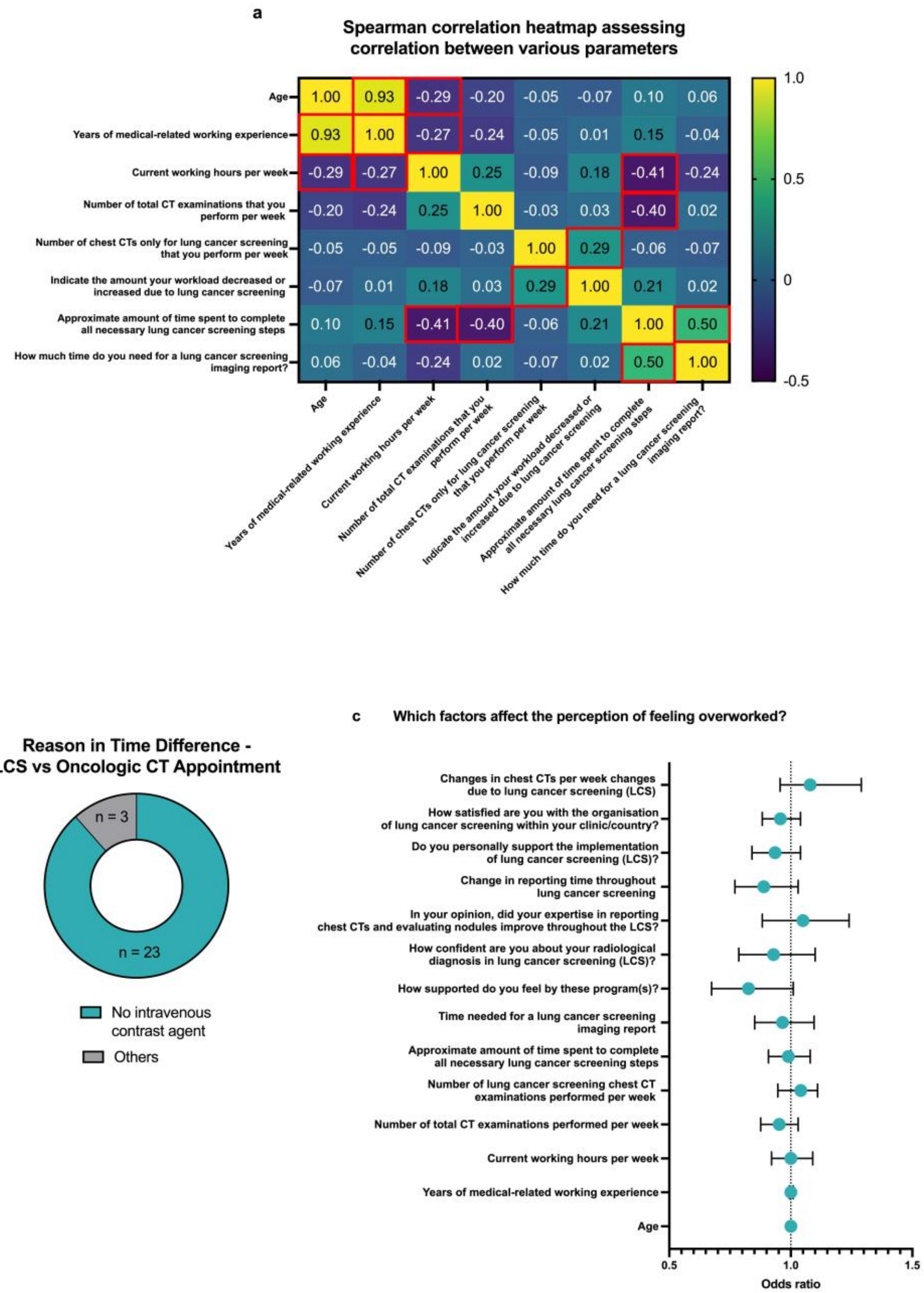

Supplementary Figure 2: Effects of Implementation of LCS on Workload.
(a): Spearman correlation heat map showing the relationship between various factors pertaining to workload. The correlation coefficient is included up to 2 decimal places. The boxes in red indicate statistically significant p-values. (b): Perceived reasons for the time difference between a LCS and an oncologic chest CT appointment. (c): Forrest plot showing the effect of selected factors on the perception of feeling overworked. The hazard ratios and 95% confidence intervals are indicated for each factor. Statistically significant factors are marked with an asterisk (*).

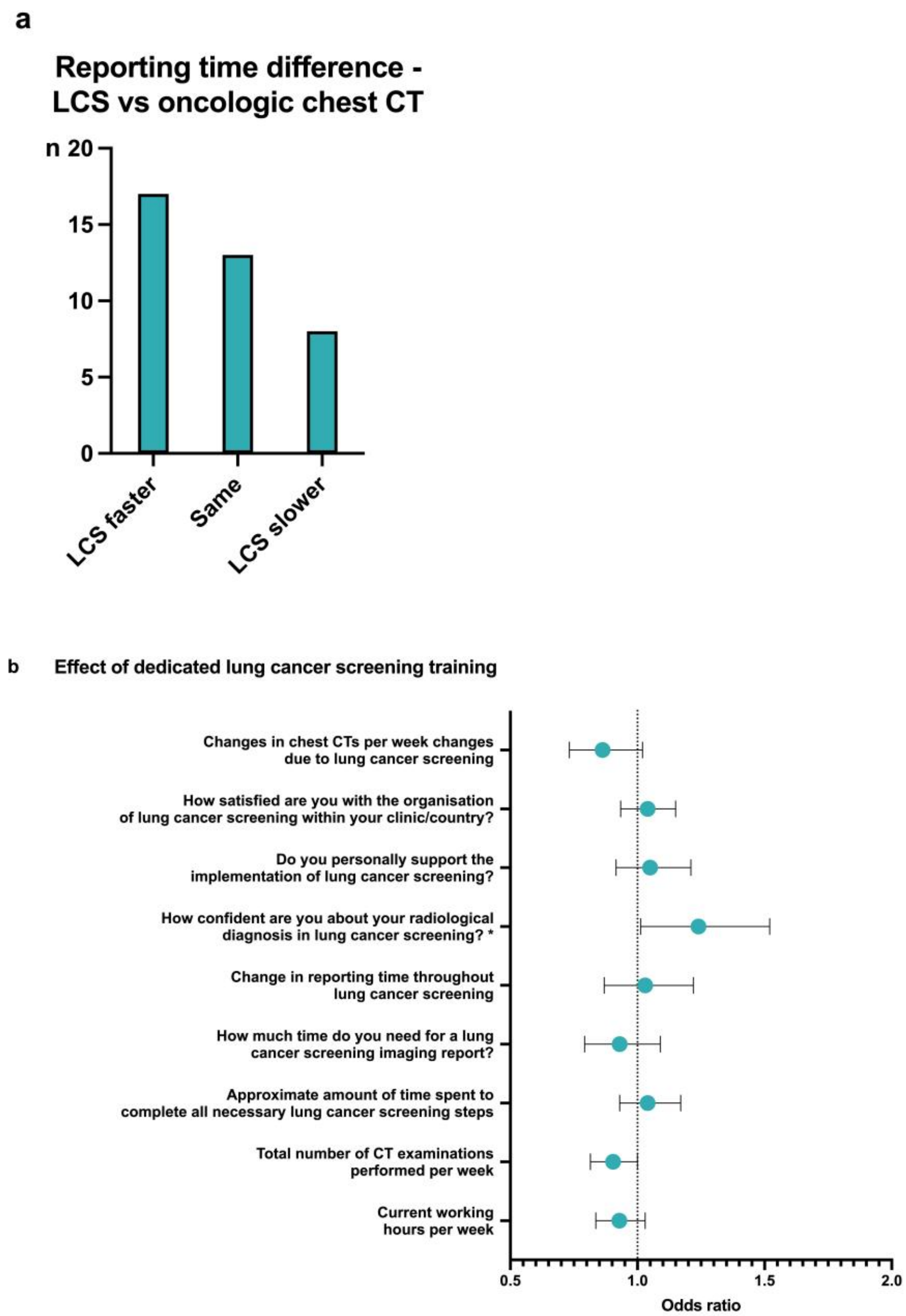

Supplementary Figure 3: Influence of the use of structured reporting, AI and LCS Training Programmes on Reporting LDCTs

(a): Difference in reporting time between an LCS and an oncologic chest CT. (b): Forrest plot showing the effect of lung cancer screening training on selected factors. The hazard ratios and 95% confidence intervals are indicated for each factor. Statistically significant factors are marked with an asterisk (*).

Supplementary Table 1. Major survey responses by occupation.

	Radiographers	Radiologists	P-value
Age (median ± SD)	46.5 ± 9.1	46 ± 11.1	0.933
Sex (male), n (%)	10 (71.4)	17 (42.5)	0.119
Years of experience (median ± SD)	18.5 ± 10.9	20, ± 11.41	0.642
Workload change due to lung cancer screening, n (%)			0.624
Decrease/no change	5 (25)	19 (43.5)	
Increase	9 (75)	21 (56.5)	
Feeling overworked due to LCS, n (%)			0.4189
Yes	1 (92.3)	9 (77.5)	
No	12 (7.7)	31 (22.5)	
Workload change due to LCS, n (%)			0.624
Decrease/no change	5 (35.7)	19 (47.5)	
Increase	9 (64.3)	21 (52.5)	
"Does your workplace conduct LCS exclusively?", n (%)			>0.999
Yes	11 (24.4)	34 (87.2)	
No	2 (28.6)	5 (12.8)	
Change in total CT due to LCS, n (%)			0.181
Decrease/no change	2 (14.3)	15 (37.5)	
Increase	12 (85.7)	25 (62.5)	
Use of automated nodule assessment programs for LCS, n (%)			0.749
Yes	6 (42.9)	14 (35)	
No	8 (57.1)	26 (65)	
Impact on level of confidence in making a correct diagnosis after using AI, n (%)			0.476
Decrease/no change	1 (33.3)	5 (17.2)	
Increase	2 (66.7)	24 (82.8)	
Change in expertise in reporting chest CTs caused by an increased number of chest CT, n (%)			0.456
Decrease/no change	1 (25)	5 (12.5)	
Increase	3 (75)	35 (87.5)	
Support for LCS, n (%)			>0.999
Yes	13 (92.9)	36 (90)	
No/neutral	1 (7.1)	4 (10)	
Satisfaction with LCS organisation at your workplace/in your home country, n (%)			0.0535
Yes	12 (85.7)	19 (47.5)	
No	2 (14.3)	21 (52.5)	
